# Samuel Hahnemann

**Published:** 1887-06

**Authors:** 


### SAMUEL HAHNEMANN,

At the recent semi-centennial of the introduction of the Homeopathic School of Medicine into New England, held in Boston, Dr. Selden H. Talcott of Middletown, N. Y., spoke as follows of " Hahnemann and His Influence Upon Modern Medicine :"

In studying the biographies of men, we find that there are those who cannot be repressed by unfavorable surroundings, or turned from a certain goal by repelling influences. The insistence of parents, and a special education, could not keep Goldsmith in a curate's gown. Much study and an expensive education in the law, could not hold Goethe from that career in letters for which he was so grandly endowed. And, though the might of poverty and the force of circumstance combined to keep Robert Burns at the plow, he found it impossible to do anything except to give voice to the melody forever in his heart. And so it was with Samuel Hahnemann. He was bound by the galling chains of poverty ; he was repelled by the scorn and ridicule of his confrères in the profession ; he was invited to other fields of labor, through his abilities as a chemist and a teacher ; but in the face of every adverse circumstance, or diverting influence he felt himself impelled to become the exponent of a new system of medical practice. And he rose with the mighty power of an inspired purpose, a clear vision, a marvelous judgment and an iron will ; and he accomplished the work to which he turned his tremendous energies.

Hahnemann not only made his mark as a chemist, and considered and understood the relationships of the material elements, but he seemed endowed with special power to pierce the veil, and to acquire a deep and true understanding of the subtle mysteries, and operations of the human soul. As a result of his clearness of vision in this direction, he formed an appreciative estimate of those products which spring from the union of soul and body, and which we call mental manifestations. Hahnemann enunciated the fact, a fact which is now generally recognized, that there are no mental diseases which do not owe their existence to a bodily disease. He also recognized the fact that bodily diseases may in turn be produced through mental avenues, such as shock from grief, or anger, or sudden excitement of any passion or emotion. Recognizing these facts, he came to the conclusion that the treatment of diseases, bodily and mental, must be based upon both a material recuperation of the physicial forces, and a spiritual tranquilizing of the mental and the moral forces.

Hahnemann placed a truer estimate upon the brain and mind than any physician who had preceded him. The ancients thought that the brain was but a useless mass of crude matter, a sort of overgrown gland, a mountain snow cap to keep the rest of the body cool. But the modern student finds, with Hahnemann, that the brain, which the ancients despised, is the chief and most important organ of the human body. The human mind, the occupant of this brain, is the marvel and the mystery of creation. It is swayed by every flitting passion or impression, and yet it is held in steady poise by the calm monitions of reason, of cultivated judgment and of developed will. In these respects it resembles those wondrous rocking stones reared by the ancient Druids. You remember that they were so finely poised that the finger of a child could vibrate them to their centres, and yet they were so firmly balanced that the might of an army could not move them from their base. So it is with the human mind, which has been thoroughly trained, carefully cultured, and kept by its owner as a pearl without price. The smile of a child can sway it to and fro, while the fagot of martyrdom could not change one jot or tittle of its firm determination.

To Hahnemann is due the honor of having been one of the great discoverers of the *sensitiveness*, and likewise the *stability*, of the human mind. And the discovery of these facts led this great physician to new and successful issues in the treatment of disease.

One hundred years ago the doctrine of a humoral pathology, under various and mystifying names, still swayed and influenced the minds of medical men. Again, the works of Galen, which had been accepted as medical scripture for thirteen hundred years, had not been utterly discarded ; and in those works we find Teachings as dark as the mysterious shadows of the Middle Ages.

Though Paracelsus had burned, with mighty ostentation, the works of Galen and of Avicenna, he failed to dispose of their influence, nor did he substitute any new or practical theory in medicine. The influence of the " great empiric " tended rather to confuse medical theories, and to encourage boastfulness and arrogance, in the practice of the healing art. Cullen had, indeed, written a materia medica, and the virtues of Peruvian bark, as a cure for intermittent fever had been discovered and partially developed. Aside from this, the medicine of Hahnemann's day, as now generally acknowledged, was a jumbled mass of chaotic theory and of bungling and unsuccessful practice. Physicians had no clear knowledge of disease itself, they had no well defined law of therapeutics, they had no thorough understanding of the nature and action of the commonest drugs. Polypharmacy everywhere prevailed. Every physician was a law unto himself. Early in the Christian era Scribonius Longus had prepared a life elixir, containing sixty-one deadly drugs, and being ignorant of the virtues of them all, he piously believed he could ladle forth healing and happiness to all mankind. The imitators of Scribonius lived in the nineteenth century, and some of them are not yet dead.

The rule of a century ago was not how little of a poisonous drug could be administered, and the cure of the patient be effected, but how much could the patient take before he died ! Herberden, in 1782, shortly after Hahnemann graduated in medicine, wrote concerning Peruvian bark, that twenty-four drachms a day for six days, providing it could be " got down," would usually weaken the fever and free the patient from danger of relapse. Fourteen hundred and forty grains of powdered Peruvian bark per day for six days in succession, was one of the prescriptions for intermittent fever a hundred years ago. Even our old school brethren some of them at least, would recoil from using so much at the present time.

The human form divine, whenever disordered, was looked upon in those days as a mass of diseased matter, and the more you could reduce the mass the more surely you could dispel the disease. Von Humboldt states that "the diseased matter is really the whole living matter itself, so far as its form and position are changed, and the balance of its elements is disturbed."

Such was the idea of disease, and such the practice of medicine, during the latter portion of the eighteenth century. About that time, however, vague attempts to change the programme were being inaugurated. The fountains of the great deep were evidently breaking up, and a series of diverse experiments were being made upon the helpless sick. Depletion was followed by stimulation, and stimulation by efforts to produce chemical changes in the general constituents of the body. The patient was knocked down by drugs, and braced up by wines, and drenched, and whitewashed, and galvanized by the various products of alchemy and chemistry. Only that which was physical and material could be examined or comprehended. Man was a species of India rubber god, wound up and turned loose to run his erratic and uncertain course. Only chemicals could affect him when sick, according to the prevailing notions of the waning century.

As a chemist Hahnemann rose to an equal eminence with any master of his time. But he was not satisfied with attempting to cure disease by so-called chemical measures. He found them inadequate to the desired ends. He investigated disease, and found it to be a disturbance of vital force, a force which is imponderable, and which cannot be evolved from any crucible of chemical art. He found that a disturbance of vital force, resulted in the impairment of normal bodily functions, and this impairment produced changes in the quality, the arrangement, and the distribution of the molecular atoms of the body. He found that diseases arise, not only through the inception of hurtful substances into the system, by means of deleterious food or drink, or by the inhalation of contaminated or bacterial air ; but he also found that diseases may arise from shock through mental impressions, and that this latter cause produces quick damage, resulting in changes in the vital forces, and in the organic structures. He found that drugs hitherto used in a blind way for the cure of disease, were capable of developing marked and positive abnormal symptoms within the hitherto healthful body. And he also found that by the use of a proven drug, in modified quantities, he could remove the symptoms of disease in a person suffering in a similar manner, as the one who had partaken of a given drug, and that, too, without first causing an aggravation. Again, he ascertained by actual experiment that the qualities of a drug may be transmitted to new vehicles, thus enabling him to use the powers of a drug with even more certainty and safety than before.

To recapitulate : Here, then, are some of the achievements of Samuel Hahnemann :

1. He portrayed the true nature of disease, and described it as a disturbance of vital force.

2. He enunciated the law of similars embodied in the doctrine "Similia, similibus, curantur"—a law upon which scientific medicine is inevitably based.

3. He inaugurated the plan of proving drugs upon the healthy before using them as medicines for the sick.

4. He discarded polypharmacy as unscientific.

5. He adopted the plan of using the single remedy, for the safe and speedy cure of disease.

6. He made war against bleeding, blistering, purging, administering emetics, and all forms of unnecessary depletion.

7. He defined medicine in a manner comprehensive enough for all time. In his Lesser Writings he states : "A knowledge of diseases, a knowledge of remedies, and a knowledge of their employment (that is, for the cure of disease), constitute medicine." That definition has not as yet been improved upon.

8. He reduced the size of the dose, until all danger of aggravation from the drug was removed. He proved the possibility of successful treatment, by the administration of medicines in minute quantities ; and when that fact was determined, there was a gradual abandonment of the "kill or cure" doses of the ancients.

9. He developed in medicine the doctrine of transmitted force, a doctrine that the inherent powers of a drug may be passed from the original material, to new material without a necessary loss of their natural energies.

Who can estimate the influence of such a man who wrought, during an eventful life, such miracle-like achievements ? He developed a philosophy as comprehensive, as beneficent, and as far-reaching in its conception of usefulnes, as the prodigious philosophies of Aristotle, of Plato, and of Lord Bacon. This man worked alone, unaided, uninspired, save by his personal sense of the possession of a mighty and glorious truth. With that truth in his soul he rose like a giant from the ranks of the people, seized the masses of antique theory and uncertain conjecture by which he found himself surrounded, and hurled them into the yawning gulf of a well-earned oblivion. He portrayed with the clearness of sunlight the folly of old-time methods of treating the sick, by rash and blindly-heroic means, and he proved the powers and effects of drugs upon himself, ere he ventured to administer them as medicines to the sick. He covered Europe with the evidences of his marvelous medical skill; he swept back the tide of long and bitter persecution by the sublime triumphs of his art; he kept up the glorious carnival of his successful practice, until he was crowned with surpassing honors in Paris; and he rested not until, by the grandeur of his achievements, the city of Leipsic, from which he had been driven as a fugitive and a vagabond, erected a stately monument to his name, a monument that remains to this day, as a fitting memorial to his magnificent and imperishable memory.

